# A Venomics Approach to the Identification and Characterization of Bioactive Peptides From Animal Venoms for Colorectal Cancer Therapy: Protocol for a Proof-of-Concept Study

**DOI:** 10.2196/31128

**Published:** 2021-12-21

**Authors:** Syeda Kiran Shahzadi, Noushad Karuvantevida, Yajnavalka Banerjee

**Affiliations:** 1 Department of Basic Medical Sciences Mohammed Bin Rashid University of Medicine and Health Sciences Dubai United Arab Emirates

**Keywords:** animal venoms, colorectal cancer, bioactive peptides, high-throughput screening, venom, cancer, colorectal, peptide, screening, treatment, conceptual, characterize, development, therapy

## Abstract

**Background:**

Cancer is the third leading cause of death in the United Arab Emirates (UAE), after cardiovascular diseases and accidents. In the UAE, colorectal cancer (CRC) is the first and fourth most common cancer in males and females, respectively. Several treatment modalities have been employed for cancer treatment, such as surgery, radiotherapy, chemotherapy, hormone replacement therapy, and immunotherapy. These treatment modalities often elicit adverse effects on normal cells, causing toxic side effects. To circumvent these toxicities, there has been an increased impetus towards the identification of alternate treatment strategies. Animal venoms are rich sources of pharmacologically active polypeptides and proteins.

**Objective:**

In this proof-of-concept study, we will apply a high-throughput venomics strategy to identify and characterize anticancer bioactive peptides (BAPs) from 20 different animal venoms, specifically targeting CRC. We chose to focus on CRC because it is one of the foremost health issues in the UAE.

**Methods:**

In the initial study, we will screen 2500 different peptides derived from 20 different animal venoms for anticancer activity specifically directed against 3 CRC cell lines and two control cell lines employing the 3-(4,5-dimethylthiazol-2-yl)-2,5-diphenyltetrazolium bromide (MTT) colorimetric assay for cytotoxicity. Of the 20 venoms, 3 that exhibit specific and potent anticancer activity directed against the 3 CRC cell lines will be selected; and from these 3 venoms, the specific peptides with anti-CRC activity will be isolated and characterized.

**Results:**

This study is at the protocol development stage only, and as such, no results are available. However, we have initiated the groundwork required to disseminate the proposed study, which includes culturing of colorectal cancer cell lines and preparation of venom screens.

**Conclusions:**

In summary, the proposed study will generate therapeutic leads to manage and treat one of the leading health issues in the UAE, namely, CRC.

**International Registered Report Identifier (IRRID):**

PRR1-10.2196/31128

## Introduction

### Background

Cancer is the third leading cause of death in the United Arab Emirates (UAE) after cardiovascular diseases and accidents. In the UAE, colorectal cancer (CRC) is the first and fourth most common cancer in males and females, respectively [1]. Numerous modalities have been employed for cancer treatment, such as surgery, radiotherapy, chemotherapy, hormone replacement therapy, and immunotherapy [2]. These treatment modalities often elicit adverse effects on normal cells, causing toxic side effects such as neurotoxicity, hepatotoxicity, and nephrotoxicity [3]. To circumvent these toxicities, there has been an increased impetus to identify alternate treatment strategies.

Animal venoms are veritable gold mines of pharmacologically active polypeptides and proteins. In fact, proteins and peptides with anticancer properties have been identified and characterized from venoms of snakes, bees, scorpions, wasps, ants, spiders, and caterpillars [4,5]. One case in point is contortrostatin, a disintegrin isolated from the venom of *Agkistrodon contortrix contortrix* (southern copperhead); although it is not cytotoxic to breast cancer cells, it inhibits angiogenesis induced by breast cancer in vivo [6]. Similarly, gonearrestide, a scorpion-derived peptide, inhibits the growth of primary colon cancer cells and solid tumors by triggering cell cycle arrest in G1 phase through inhibition of cyclin‐dependent kinase 4 and by the upregulation of the expression of cell cycle regulators and inhibitors—cyclin D3, p27, and p21 [7]. Furthermore, conopeptides derived from *Conus inscriptus* have been shown to possess prospective anticancer activity, specifically against cervical cancer [8].

In summary, animal venoms comprise a mix of bioactive peptides (BAPs), many of which exhibit anticancer activity against specific types of cancer mediated by one of the following four mechanisms: (1) induction of cell cycle arrest, growth inhibition, and apoptosis; (2) inhibition of angiogenesis; (3) inhibition of invasion and metastasis; and (4) blocking of specific transmembrane channels [9].

In this proof-of-concept study, we will apply a high-throughput venomics strategy to identify and characterize anticancer peptides from 20 different animal venoms, specifically targeting colorectal cancer. We chose to focus on colorectal cancer because it is one of the foremost health issues in the UAE.

The proof-of-concept study will address two specific aims.

In Aim 1, we will screen 2500 different peptides derived from 20 different animal venoms for anticancer activity specifically directed against 3 CRC cell lines and 2 control cell lines employing the 3-(4,5-dimethylthiazol-2-yl)-2,5-diphenyltetra- zolium bromide (MTT) colorimetric assay for cytotoxicity.

In Aim 2, 3 venoms of the 20 screened in Aim 1 that exhibit specific and potent anticancer activity directed against the 3 CRC cell lines will be selected; from these 3 venoms, the specific peptides with anti-CRC activity will be isolated and characterized.

## Methods

**Figure 1:**
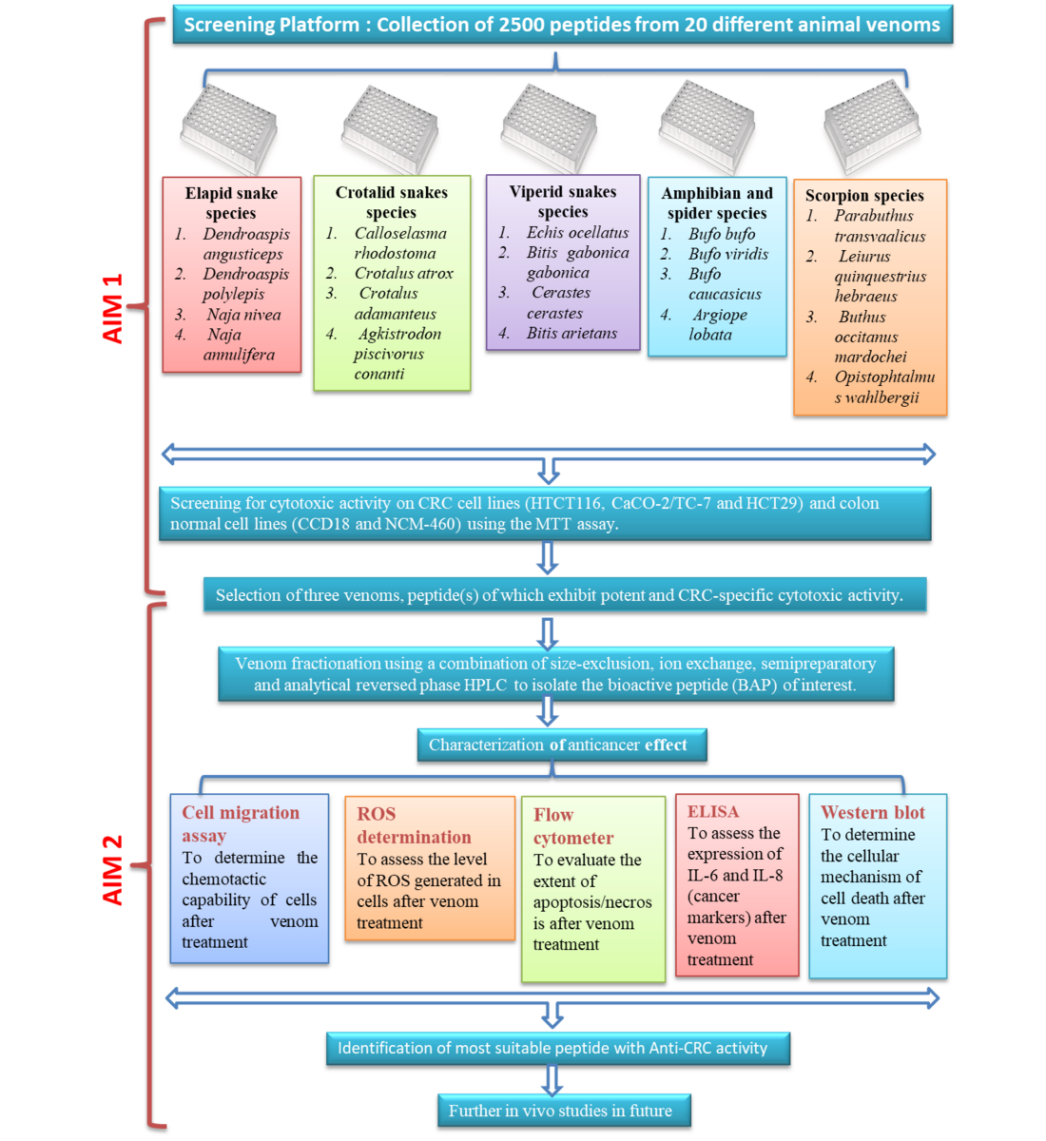
Dissemination plan for the proposed research. CRC: colorectal cancer; HPLC: high-performance liquid chromatography; ROS: reactive oxygen species.

The dissemination plan for the proposed research corresponding to Aim 1 and Aim 2 is shown in Figure 1.

### Aim 1: Dissemination

Aim 1 of the study is directed to the identification of three animal venoms which exhibit potent and specific cytotoxic activity on CRC cell lines (HCT116, Caco-2/TC-7, and HT29) and normal colon cell lines (CCD18 and NCM-460).

#### Preparation of the Venom-Derived Peptide Screen

This step of the project will be pursued in collaboration with Latoxan (Valence, France), a leading producer of animal venoms, with whom we have engaged in previous research collaborations. Briefly, a library of venom peptides will be created in 96-well plates. Venoms will be first cleared from molecules over 8500 Da (cytolytic enzymes). On the basis of high-performance liquid chromatography (HPLC), each venom will be split into 20 fractions. Each fraction is expected to contain 5 to 10 peptides at a 10 μmolar concentration in a volume of 100 μl. The strength of this strategy is that the separation of peptides using HPLC facilitates the identification of the active peptide once a hit (specific cytotoxic activity on CRC cell lines) is detected. Each 96-well plate will be filled with the fractions derived from 4 venoms, equaling 80 fractions and approximately 500 different peptides in total. Therefore, the entire screen contains 2500 venom-derived peptides separated in 400 lyophilized fractions from 20 different animal venoms (refer to Figure 1 for details of the species that will be included in the screen).

#### Cell Culture

Human colorectal cancer cell lines (HCT116, Caco-2/TC-7, and HT29) and normal colon cell lines (CCD18Co and NCM-460) will be purchased from American Type Culture Collection. For cell culturing, RPMI 1640 (Sigma-Aldrich) medium will be used, supplemented with 10% fetal bovine serum (Sigma-Aldrich) and 1% penicillin/streptomycin antibiotic cocktail (Sigma-Aldrich). The culture flasks (T25/T75) will be incubated in a 5% CO_2_ humidiﬁed incubator at 37 ºC. After reaching 70% to 80% confluency, the cells will be split using 1% trypsin–ethylenediaminetetraacetic acid (EDTA) solution (Sigma-Aldrich).

#### Cytotoxicity on Normal and CRC Cell Lines

High-throughput screening of 400 lyophilized venom fractions from 20 different animal species will be executed by measuring the cytotoxic activity of the fractions on CRC cell lines (HCT116, Caco-2/TC-7, and HCT29) and normal colon cell lines (CCD18 and NCM-460) using the [3-(4,5-dimethylthiazol-2-yl)-2,5-diphenyltetrazolium bromide (MTT) assay (Merck Millipore) using the methods depicted in [10,11]. First, the lyophilized fractions will be diluted in dimethyl sulfoxide (DMSO) for further experiments. Secondly, 3000 to 4000 cells/well will be grown in 96-well plates, and after 24 hours, the cells will be introduced to 2 μM concentrations of venom fractions in complete culture medium, maintaining a blank (no venom) as a negative control and 5-fluorouracil as a positive control. After 72 hours of incubation, the cells will be incubated with 10% MTT, followed by 3 hours of incubation in dark in a 5% CO_2_ humidiﬁed 37 ºC incubator. The purple formazan formed will be dissolved in DMSO (Sigma-Aldrich), and the plates will be quantified by measuring the absorbance at 570 nm using a microplate reader (Hidex). The percent viability of the cells will be calculated as:

[(absorbance of experimental sample) – (absorbance of blank sample)] / [(absorbance of untreated sample) – (absorbance of blank sample)] × 100

IC_50_ values and statistical significance will be determined using one-way analysis of variance.

#### Expected Results

For 3 animal venoms, fractions (containing peptides) that exhibit specific and potent cytotoxic activity on CRC cell lines will be selected and used for subsequent studies, as depicted in Aim 2. We aim to focus on peptides; therefore, during the preparation of the screen, we will eliminate the cytolytic enzymes in the venoms.

### Aim 2: Dissemination

#### Purification of BAPs With CRC Cell Line–Specific Cytotoxic Activity From the 3 Venoms Identified in Aim 1

The 3 venoms identified in Aim 1 will be procured in bulk from Latoxan. BAPs with CRC cell line–specific cytotoxic activity will be purified from the venoms using a combination of size exclusion, ion exchange, and semipreparatory and analytical reverse-phase high-performance liquid chromatography on an AKTA Avant multidimensional chromatography system (GE Healthcare Systems) that is available in our laboratory. The purification strategy will be similar to that described by Banerjee et al [12,13]. At each step of purification, we will check for cytotoxic activity, employing the MTT assay to identify the fraction in which the BAP is present. The purified BAP fractions will be collected, lyophilized, and stored at −20 °C until further use.

Under Aim 1, once we have screened all the venom fractions, we will analyze the hits that we have achieved. For this part of the project, we will be focusing on the hits for which cytotoxic activity is specifically observed against HT-29 cells. We will select the 3 venoms corresponding to the peptides that exhibit the most potent cytotoxic activity specifically against HT-29 cells. The remaining information corresponding to the peptides that exhibit cytotoxic activity will be used to create a biobank of active peptides, which will be the focus of later studies.

Therefore, under Aim 2 of the study, we will focus on purifying BAPs from the 3 venoms that we have selected following the screening process. These BAPs will be purified to homogeneity using a combination of chromatographic techniques (as indicated above). These purified peptides will be further characterized to identify the molecular mechanism by which they mediate the anticancer activity. Because the characterization phase will require BAPs in significant quantities, we also will use fluorenylmethoxycarbonyl (Fmoc) solid phase peptide synthesis for bulk synthesis of the BAPs. In a later phase of the study, we will establish a bacterial expression system for the mass production of the BAPs identified and characterized in Aim 2 using the methodology depicted in [14].

#### Determination of the Molecular Weight of the BAPs

The homogeneity and molecular weight of the BAPs will be determined by electrospray ionization mass spectrometry using a PerkinElmer Life Sciences API-300 liquid chromatography/tandem mass spectrometry (MS) system at the MS facility at SGS (Geneva, Switzerland). Typically, reverse-phase HPLC fractions will be directly used for analysis. The ion spray, orifice, and ring voltages will be standardized for analysis. Nitrogen will be used as a nebulizer and curtain gas. A LC-10AD pump (Shimadzu) will be used for solvent delivery (40% acetonitrile in 0.1% trifluoroacetic acid) at a flow rate of 50 μl/min. BioMultiview software (PerkinElmer Life Sciences) will be used to analyze and deconvolute the raw mass spectra.

#### Reduction and Pyridylethylation

The purified BAPs will be reduced and pyridylethylated using procedures described previously. Briefly, BAPs (0.5 mg) will be dissolved in 500 μl of denaturant buffer (6 m guanidine hydrochloride, 0.25 m Tris-HCl, and 1 mm EDTA [pH 8.5]). After the addition of 10 μl of β-mercaptoethanol, the mixture will be incubated under vacuum for 2 hours at 37 °C. 4-Vinylpyridine (50 μl) will be added to the mixture, and it will be maintained at room temperature for 2 hours. The pyridylethylated BAPs will be purified on a Jupiter C18 analytical column (4.6×250 mm) using a gradient of acetonitrile in 0.1% (volume/volume) trifluoroacetic acid at a flow rate of 0.5 ml/min.

#### N-Terminal Sequencing

N-terminal sequencing of the native and S-pyridylethylated BAPs will be performed by automated Edman degradation using a pulsed liquid-phase sequencer (PerkinElmer Life Sciences Model 494, Procise) with an online Model 785A phenylthiohydantoin derivative analyzer (Applied Biosystems).

### Characterization of the Anticancer Properties of the BAPs

Using a combination of different in vitro experiments, the anticancer properties of the BAPs will be characterized. The experimental protocols and the rationale for pursuing these experiments are indicated below.

#### Cell Migration Assay

Cell motility is a very important parameter in determining the survival and progression of cancer. Augmented cancer cell motility is the root cause of end-stage organ damage causing mortality [15]. The cell motility assay will be performed according to a procedure recommended by Rodriguez et al [16]. Briefly, CRC cells will be grown in a 60 mm Petri dish until they reach 80% confluency; then, a fine scratch will be made with the aid of a sterile pipette tip, and the scratch will be immediately photographed at hour 0. Next, cells will be supplemented with complete medium to allow them to grow. After 24 hours, the migration of the cells from the scratched area will be monitored microscopically. The width of the scratch at 0 and 24 hours will be measured, and the percentage of the gap covered by the cells will be calculated.

#### Determination of Reactive Oxygen Species Levels

Cancer metastasis involves a slight elevation in the production of reactive oxygen species (ROS). Cancer cells appear to thrive on high levels of ROS compared to their normal counterparts, as cancer cells have developed augmented antioxidant systems [17]. ROS generation in CRC cells and normal cells after in the presence and absence of BAPs will be assessed using 2,7-dichlorofluorescein diacetate (DCFH-DA) (Sigma-Aldrich) using the manufacturer’s protocols. Briefly, CRC cells and normal cells will be plated in 6-well plates (0.3 million cells/well), and subsequently, subconfluent cells will be treated with BAPs for half an hour. Afterwards, the cells will be trypsinized and plated in new black 96-well plates, followed by incubation with 10 μM DCFH-DA for 4 hours at 37 °C. The fluorescence measurements will be performed in a Hidex microplate reader (Turku) at an excitation and emission wavelength of 485 nm and 538 nm, respectively.

#### Investigating Apoptosis Using Flow Cytometric Experiments

The extent of apoptosis caused by BAP will be evaluated on a FACSAria III flow cytometer (BD Biosciences) using an annexin V and 7-aminoactinomycin D (7-AAD) apoptosis detection kit (BioLegend). Cells at a concentration of 0.3 M will be seeded in a 6-well plate and incubated in a 37 ºC 5% CO_2_ humidiﬁed incubator. After 24 hours, the cells will be treated with benzopyrene and 5-fluorouracil (positive control) for 24 hours. Cells will be stained with annexin V and 7-AAD in the dark for 20 minutes according to the manufacturer’s protocols. FlowJo 10.7.1 software (BD Biosciences) will be used to analyze the data.

#### Enzyme-Linked Immunosorbent Assay

Elevated levels of vascular endothelial growth factor, IL-6, and IL-8 are proven biological markers indicating cancer progression [18,19]. Taking into consideration the proactive character of these cytokines (IL-8 and IL-6) in cancer, we will assess their expression in CRC cell lines and normal cell lines following BAP treatment using the method described by Duffy et al [18]. Briefly, 0.3 million cells will be seeded in complete culture medium containing 1% bovine serum albumin (BSA) and incubated in a 37 ºC 5% CO_2_ humidiﬁed incubator for 6 hours. Purified BAPs will be introduced to the cells, followed by incubation for 24 hours. After 24 hours, the cell culture media will be collected and centrifuged to remove the cell debris. The adherent cells will be collected after trypsinization and will be counted to normalize the cytokine concentration. The assay will be executed as per the manufacturer’s guideline on enzyme-linked immunosorbent assay (ELISA) plates (ExtraGene) precoated with human IL-6 and IL-8. The colorimetric intensity will be measured on a microplate reader (Hidex) at 450 nm.

### Western Blot Analysis

RhoC is a metastatic protein that is found to be constitutively active in many types of cancers, including CRC [20]. We will check the expression level of RhoC and the phosphorylation of its downstream targets, ERK1/2, JNK, and P38, in BAP-treated cells to investigate the disruption of the signaling mechanisms. Equal amounts of proteins extracted from control and BAP-treated cells will be loaded on 12% sodium dodecyl sulfate-polyacrylamide gel using a Mini-PROTEAN system (Bio-Rad Laboratories). Proteins will be transferred to nitrocellulose membranes using an iBlot 2 gel transfer device (Thermo Fisher Scientific). The membranes will be incubated with RhoC, ERK1/2, JNK, P38, and GAPDH antibodies (Abcam) after blocking of the membranes with 3% BSA. After the membranes are incubated with secondary antibodies, the bands will be visualized on a ChemiDoc MP Imaging System (Bio-Rad Laboratories) with enhanced chemiluminescence detection reagents.

### Statistical Analysis

Statistical analyses will be accomplished using the Student *t* test in Stata (StataCorp LLC). The mean values will be reported with standard deviations. *P* values ≤.05 will be considered significant for differences.

## Results

This study is at the protocol development stage, and as such, no results are available. Experimental procedures in this study will be conducted in vitro and will not involve the use of animal models or samples, patient samples or data, or recruitment of human subjects. Therefore, research conducted as part of this study poses minimal risk and fits one of the exempt review categories as defined by institutional review board (IRB) regulations at Mohammed Bin Rashid University (MBRU). Further clarification and information can be obtained from the MBRU IRB at irb@mbru.ac.ae. We received funding for this study following review of our proposal. The funding ID for our research is MBRU-CM-RG2021-08. Additionally, we have initiated the groundwork for this study, which includes the purchase of CRC cell lines and preparation of the venom screen kit in collaboration with Latoxan.

## Discussion

Animal venoms are cocktails of pharmacologically active polypeptides and proteins. Therapeutic leads from venom have been successfully developed into drugs, such as the following examples.

### Captopril

This drug [21] was developed based on the therapeutic lead of teprotide, identified and characterized from the Brazilian arrowhead viper (*Bothrops jararaca*). Captopril is a potent hypotensive agent used to manage/treat hypertension.

### Tirofiban

This peptidomimetic drug [22] was first approved for therapeutic use in 1998; the original peptide lead was isolated and characterized from the venom of the African saw-scaled viper (*Echis ocellatus*). Tirofiban is commonly prescribed to patients recovering from heart attacks or experiencing angina.

### Ziconotide

Ziconotide [23] is a peptidomimetic drug sold under the brand name Prialt; the original peptide was identified and characterized from the venom of *Conus magus*. Ziconotide is injected into spinal fluid to prevent pain signals from reaching the brain.

Our studies will identify novel BAPs with anti-CRC activity. These BAPs will act as therapeutic leads for the development of peptidomimetics; in the initial stages, we will apply a similar strategy to that depicted by Al-Amri et al [24] for the development of the peptides, which will have future potential for commercialization. Therefore, in the proposed study, we will attempt to develop a sufficient and resilient infrastructure capable of supporting anticipated economic growth in the UAE. Additionally, through the dissemination of this project, we will endeavor to train 7 undergraduate students and 1 graduate student in the niche of biomedical research. This aspect attests to the development of a highly skilled productive research workforce in the UAE.

Several studies are available in the literature in which the anticancer properties of animal venoms specifically targeting CRC have been investigated. However, these studies mostly focus on the anticancer properties of the whole venom rather than on the isolation and characterization of BAPs mediating the anticancer effect. For example, in the study by Al-Asmari et al [25], the anti-colorectal cancer properties of three scorpion venoms, *Androctonus bicolor*, *Androctonus crassicauda*, and *Leiurus quinquestriatus*, were assessed, but the study fell short of identifying the specific BAPs that mediated the anticancer activity. Although such studies are of biochemical research interest, they are not therapeutically viable, as whole venom cannot be administered for treating patients with CRC. Our study, on the other hand, presents a rational approach in which we will first screen for anti-CRC of specific venom, followed by isolation and characterization of those BAPs that exhibit these activities with high potency. Therefore, the strategy applied in this study can also be adopted for similar studies intending to isolate and characterize BAPs from different animal venoms.

## Abbreviations

7-AAD: 7-aminoactinomycin D
BAP: bioactive peptide
BSA: bovine serum albumin
CRC: colorectal cancer
DCFH-DA: 2,7-dichlorofluorescein diacetate
DMSO: dimethyl sulfoxide
EDTA: ethylenediaminetetraacetic acid
ELISA: enzyme-linked immunosorbent assay
Fmoc: fluorenylmethoxycarbonyl
HPLC: high-performance liquid chromatography
IRB: institutional review board
MBRU: Mohammed Bin Rashid University
MS: mass spectrometry
MTT: 3-(4,5-dimethylthiazol-2-yl)-2,5-diphenyltetrazolium bromide
ROS: reactive oxygen species
UAE: United Arab Emirates
